# Ideal Levels of Prosocial Involvement in Relation to Momentary Affect and Eudaimonia: Exploring the Golden Mean

**DOI:** 10.1093/geroni/igaa057.2083

**Published:** 2020-12-16

**Authors:** Kelsey Finley, Maria Axner, Katherine Vrooman, Dwight Tse

**Affiliations:** 1 Claremont Graduate University, Claremont, California, United States; 2 The Chinese University of Hong Kong, Hong Kong, China

## Abstract

Aristotle believed that happiness and success result from cultivating virtue at the mean between deficiency and excess - the golden mean. Some evidence suggests there is a golden mean of hours spent volunteering, where well-being benefits are maximized. Our study examined potential linear and nonlinear functions in the amount of time spent in prosocial work (PSW) in a day on the outcomes of eudaimonia, high arousal negative affect, and high arousal positive affect in a sample of high-commitment volunteers and prosocial leaders. In addition to nonlinear functions, interactions were explored. For example, we found that those who spend less time per week in PSW experience less negative affect when they spend more hours per day in PSW; however, those who spend more time per week in PSW experience more negative affect when they spend more hours per day in PSW. Additional findings for positive affect and eudaimonia will be discussed.

